# Langerhans cell histiocytosis of the thyroid complicated by papillary thyroid carcinoma: A case report and brief literature review

**DOI:** 10.1097/MD.0000000000007954

**Published:** 2017-09-01

**Authors:** Xin Wu, Shi Chen, Li-yang Zhang, Ya-ping Luo, Ying Jiang, Rui-e Feng

**Affiliations:** a Department of General Surgery; b Department of Endocrinology; c Department of Nuclear Medicine; d Department of Pathology, Peking Union Medical College Hospital, Chinese Academy of Medical Sciences and Peking Union Medical College, Beijing, China.

**Keywords:** Langerhans cell histiocytosis, papillary thyroid carcinoma, surgery

## Abstract

**Rationale:**

Langerhans cell histiocytosis (LCH) involves mainly the skin and bone and rarely the thyroid. Meanwhile, papillary thyroid carcinoma (PTC) is the most common subtype of thyroid cancer. Both LCH and PTC could make the thyroid enlarged and hypermetabolic. The coincidence of these 2 events in a patient is rare, and this paper aimed to report such case.

**Patient concerns:**

A 40-year-old man presented with polyuria and polydipsia for 5 years. The symptoms had been relieved well by drug therapy for >4 years, until the drugs could not control the symptoms anymore and an extensively enlarged thyroid gland was noticed.

**Diagnoses:**

Thyroid ultrasound showed a nodule with microcalcification in the upper right lobe, positron emission tomography/computer tomography scan demonstrated thyroid hypermetabolism, and fine needle aspiration (FNA) revealed PTC. Right lobectomy of the thyroid and cervical lymph node biopsy verified the diagnosis “LCH of the thyroid complicated by PTC.”

**Interventions:**

The ultrasound-guided FNA biopsy was performed prior to right lobectomy of the thyroid and cervical lymph node biopsy. Postoperative histopathological examination confirmed the diagnosis, after which the patient received adjuvant chemotherapy.

**Outcomes:**

After 5 cycles of adjuvant chemotherapy, the patient had been followed up for 2 years. LCH was controlled satisfactorily and there was no significant sign of recurrence or metastasis of PTC.

**Lessons:**

LCH of the thyroid complicated by PTC is rare. Thyroid involvement should always be considered in the differential diagnosis of LCH patients. Surgery for PTC followed by chemotherapy for LCH may be the suitable treatment.

## 1 Introduction

Langerhans cell histiocytosis (LCH) is a rare disease characterized by antigen-presenting Langerhans cells. It could involve several organs and systems such as the skin, lung, bone, liver, lymph nodes, hypothalamus, and central nervous system.^[1–3]^ The exact etiology is still under investigation.^[4,5]^ Differentiated thyroid cancer, especially papillary thyroid carcinoma (PTC), is the most common subtype of thyroid cancer, having an increasing incidence worldwide, possibly due to the increasing use of diagnostic imaging. LCH of the thyroid complicated by PTC is extremely rare. To our knowledge, reports of such case are very few.^[3,6,7]^ This paper aimed to report such a rare case and discuss its clinical features.

## 2 Case presentation

A 40-year-old Han Chinese man presented with polyuria and polydipsia for 5 years. He had to drink >5 L of water per day and was diagnosed with physiologic polydipsia. The symptoms had been relieved well by hydrochlorothiazide and carbamazepine for >4 years, until the drugs could not control the symptoms anymore and an extensively enlarged, painless, and diffusely firm thyroid gland was noticed. The patient came to Peking Union Medical College Hospital for consultation. Physical examination revealed 3rd-degree thyroid gland enlargement. The patient also had malaise and hypaphrodisia but denied having diabetes, nephritis, lethargy, bone and arthrosis pain, and skin lesions. There was no significant family history of psychiatric disorders or cancers.

**Figure 1:**
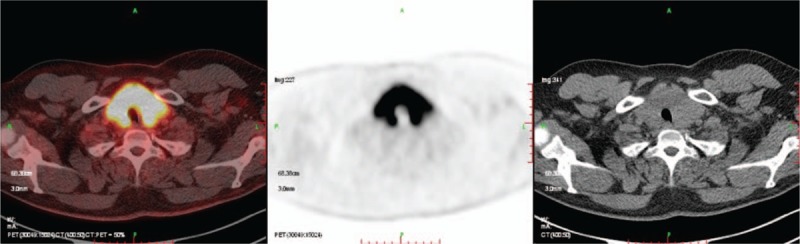
Positron emission tomography/computer tomography (PET/CT) showed extensively abnormal hypermetabolism in the thyroid. The unusual lesion might be caused by Langerhans cell histiocytosis; however, papillary thyroid carcinoma could not be excluded.

Thyroid ultrasound showed a 2.9 × 3.6 × 1.8 cm nodule with microcalcification in the upper right lobe and abnormally thickened cervical lymph nodes on the right side. Chest and abdomen computer tomography (CT) showed numerous miliary nodules in the right and left lungs and uneven low-density lesions in the liver. Cephalic magnetic resonance imaging showed a 1.3 cm lesion in the hypothalamus. Radionuclide whole-body bone imaging and thyroid functional test were normal. The patient underwent positron emission tomography/CT, which showed abnormal hypermetabolism in the saddle area, lungs, palate, thyroid, and cervical lymph nodes (Fig. 1).

**Figure 2:**
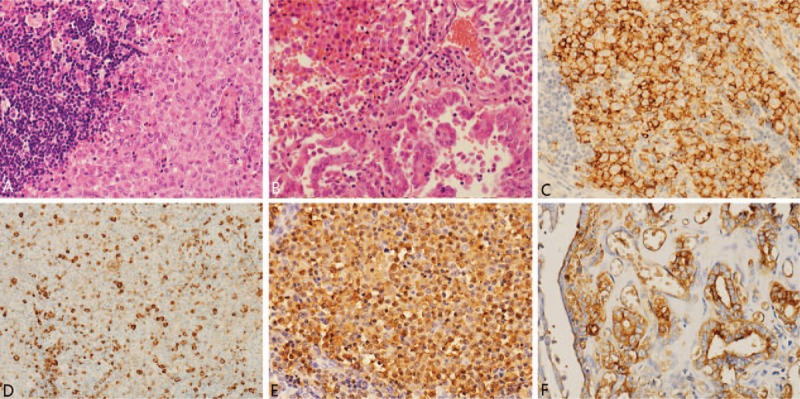
Slide description. (A) Hematoxylin and eosin image of the lymph nodes (×20) revealed LCH. (B) Hematoxylin and eosin image of the thyroid (×20) revealed LCH complicated by PTC. (C–F) Immunohistochemistry of CD1a (C, ×20), CD68 (D, ×20), S100 (E, ×20), and Thy (F, ×20) showed positive results that revealed LCH complicated by PTC. LCH = Langerhans cell histiocytosis, PTC = papillary thyroid carcinoma.

The systemic symptoms and test results could be attributed to LCH, which was confirmed pathologically by palate biopsy. However, the lesion in the thyroid could be either LCH or PTC, just as the positron emission tomography/CT scan revealed. Ultrasound-guided fine needle aspiration (FNA) biopsy of the thyroid was performed; the pathological result revealed PTC. Thereafter, right lobectomy of the thyroid and cervical lymph node biopsy were performed. Frozen section of the lymph node showed LCH involvement. No complication was observed and the patient was discharged 5 days after surgery. Finally, the postoperative pathological test verified the diagnosis “LCH of the thyroid complicated by PTC” (Fig. 2).

The patient was transferred to the hematology department and received chemotherapy containing methotrexate and cytosine arabinoside. The side effect was diarrhea, of which the patient was able to recover from. After 5 cycles of adjuvant chemotherapy, the patient had been followed up for 2 years. Thyroid ultrasound, CT, and blood tests were performed to evaluate the disease condition. LCH was controlled satisfactorily and there was no significant sign of recurrence or metastasis of PTC.

## 3 Discussion

The incidence of LCH is about 4.0 to 5.4 in 1,000,000 individuals annually.^[4]^ LCH is more common in children and male patients. The male-to-female ratio is 3.7:1.^[3]^ The clinical features depend on the involved organs or systems. Due to the various areas of lesions and complex manifestations, misdiagnosis is usual. The skeletal system, especially the skull, is the most common system involved in LCH. There could be no significant symptoms except pain and swelling. The skin is also easily involved; however, the exact incidence is unknown because most skin lesions are hardly noticeable. In the endocrine system, the hypophysis has the highest known incidence of LCH.^[8]^

LCH does not typically involve the thyroid. Most cases involving the thyroid are from case reports or retrospective studies with small samples. Thyroid involvement is more prevalent in adults. The male-to-female ratio is 1:1.4. Thyroid ultrasonography has shown diffuse lesions in about 59% of patients, nodular enlargement in 25.8%, and uneven enlargement in 13.6%.^[4]^ Nodules of calcification are found in about 10% of patients.^[9]^ In this study's case, the thyroid appeared as a diffuse enlargement.

LCH involving the thyroid could coexist with other thyroid diseases such as chronic lymphocytic thyroiditis and PTC.^[3]^ The presence of these other diseases could increase the difficulty of diagnosing LCH involving the thyroid. Thus, in patients with LCH, especially involving several organs and systems, thyroid involvement should always be considered in the differential diagnosis. In this regard, core needle biopsy of the thyroid plays an important role.^[9,10]^ The thyroid is much more operable, and the procedure is safer when performed in the thyroid than in the liver or hypophysis. However, the diagnosis could sometimes be segmental due to the limited biopsy tissue. Moreover, FNA cytological diagnosis is difficult because LCH is easily confused with other diseases such as lymphocytic thyroiditis, undifferentiated carcinoma, and lymphoma.^[11]^ LCH also shares some cytomorphologic features with PTC, for example, grooved nuclei, which makes the pathological diagnosis even more difficult.^[12,13]^ Another interesting feature of LCH involving the thyroid is the much less involvement of bone than in LCH involving other organs or systems.^[9]^

Surgery, radiotherapy, and chemotherapy can be used for treating LCH involving the thyroid,^[4]^ with chemotherapy being the most important. The traditional and classical chemotherapy regimen is vincaleukoblastinum or etoposide combined with prednisone.^[14]^ Vemurafenib has been used in some LCH patients with *BRAF* gene mutation.^[15]^ LCH shows a close association with neoplasms. Lymphoma, leukemia, and lung cancer are the top 3 that have been described, and thyroid localization may be the 4th.^[6]^ PTC has the most favorable overall prognosis of all thyroid carcinomas. Metastases are mostly confined to cervical lymph nodes, and distant metastases are very rare. The standard treatment for PTC is surgery followed by either radioactive iodine therapy or observation. The staging system depends on age, with 45 years as the threshold.^[16]^ It is not so common to find both PTC and LCH in one patient's thyroid, but if this happens, the treatment strategy will be different.

In this study's case, the diagnosis of LCH was based on palate biopsy and clinical symptoms. The difficulty was in determining whether the cause of thyroid enlargement was LCH or PTC. Both could present as an enlarged thyroid gland or nodules. However, the recommended treatments were different. Chemotherapy was the first choice if the diagnosis was LCH and if multisystem involvement had been confirmed and no cervical compression symptoms existed. In contrast, surgery was to be performed unquestionably if the diagnosis was PTC. The ultrasound-guided FNA biopsy of the thyroid revealed PTC; thus, right lobectomy of the thyroid was performed. However, the frozen section of cervical lymph node showed LCH and the single PTC nodule was well encircled by normal thyroid tissue; hence, the left lobe was spared to avoid operative complications. Chemotherapy was given after surgery and produced a satisfactory effect.

Surgery is a challenge in the treatment of LCH of the thyroid complicated by PTC. Because of the intumescent and immobile nature of the thyroid, several complications could occur after thyroidectomy. Therefore, whether the operation should be performed and what is the ideal scope of operation become controversial.^[1,17]^ If LCH is isolated in the thyroid, surgery should be the gold standard treatment whether or not PTC exists. If LCH involves several organs or systems, just as it usually does, cervical compressive symptoms and the circumstances of PTC should be assessed carefully. The compressive symptoms should be relieved by surgery, and total thyroidectomy should even be performed if necessary.^[17]^ PTC should be treated without the interference of LCH treatment. However, if PTC has only one lesion and cervical lymph nodes are negative, as in this case, total unilateral thyroid lobectomy may be a good choice. In patients with LCH and PTC, an enlarged and symptomatic thyroid is typical.^[6]^ This could be the reason why surgical excision is frequently chosen even though intraoperative hemorrhage and adjacent organ damage are common.

## 4 Conclusion

LCH of the thyroid complicated by PTC is rare. Both conditions require pathological evidence to confirm diagnosis. Surgery for PTC followed by chemotherapy for LCH may be the suitable treatment.

## Acknowledgment

The authors thank the patient for his cooperation in the follow-up and for allowing to report this case.
